# 

**Published:** 1904-06

**Authors:** 


### The Library of the Bristol Medico-Chirurgical Society.

*The following donations have been received since the publication of the List in March :*

*May 31st, 1904.*

| | |
|---|---|
| The Alumni Association of the Albany Medical College (1) ... ... ... ... ... ... ... ... | 1 volume. |
| The American Gynecological Society (2) ... ... | 11 volumes. |
| The British Balneological and Climatological Society (3) ... ... ... ... ... ... ... ... | 1 volume. |
| Cassell and Company (4) ... ... ... ... ... ... | 1 ,, |
| L. M. Griffiths (5) ... ... ... ... ... ... ... | 1 ,, |
| The Laryngological Society of London (6) ... ... | 1 ,, |
| National Association for the Prevention of Consumption (7)... .. ... ... ... ... ... ... | 2 volumes. |
| Pennsylvania State Medical Society (8)... ... ... | 1 volume. |
| Providence (R.I.) Medical Association (9) ... ... | 1 ,, |
| Rhode Island Medical Society (10)... ... ... ... | 2 volumes. |
| Sir James Sawyer, M.D. (11) ... ... ... ... ... | 1 volume. |
| The Scholastic Trading Company (12) ... ... ... | 1 ,, |

Unbound periodicals have been received from Dr. R. Shingleton Smith; and Dr. J. O. Symes has presented some unframed portraits.

### TRANSACTIONS, REPORTS, JOURNALS, &c.

Albany Medical Annals ... ... ... ... ... ... ... (1) Vol. XXIV. 1903
Alienist and Neurologist, The ... ... ... ... ... ... Vol. XXIV. 1903
American Gynecological Society, Transactions of the (2) Vols. IV.–VIII. 1879-83
American Journal of Obstetrics, The ... ... ... ... Vol. XLVIII. 1903
American Journal of Ophthalmology, The ... ... ... ... Vol. XX. 1903
American Journal of the Medical Sciences, The, Vols. CXXV., CXXVI. 1903
American Medicine ... ... ... ... ... ... ... ... ... ... Vol. VI. 1903
American Surgical Association, Transactions of the... ... Vol. XXI. 1903
Archives de Neurologie ... ... ... ... ... ... ... Tom. XV., XVI. 1903
Archives of Pediatrics ... ... ... ... ... ... ... ... ... Vol. XX. 1903
Archives provinciales de Chirurgie ... ... ... ... ... Tom. XII. 1903
Australasian Medical Gazette, The ... ... ... ... ... Vol. XXII. 1903
Birmingham Medical Review, The ... ... ... ... ... N.S., Vol. I. 1903
Bookseller, The ... ... ... ... ... ... ... ... ... ... ... ... ... ... 1903
Boston Medical and Surgical Journal, The... ... ... Vol. CXLIX. 1903
Bristol Medico-Chirurgical Journal, The ... ... ... ... Vol. XXI. 1903
British Journal of Dermatology, The ... ... ... ... ... Vol. XV. 1903
Bulletin de l'Académie de Médecine ... ... ... ... Tom. XLIX., L. 1903
Bulletin de l'Académie royale de Médecine de Belgique ... Tom. XVII. 1903
Canadian Practitioner and Review, The ... ... ... Vol. XXVIII. 1903
Cincinnati Lancet-Clinic, The ... ... ... ... ... ... ... Vol. XC. 1903
College of Physicians of Philadelphia, Transactions of the Vol. XXV. 1903
Echo médical du Nord, L' ... ... ... ... ... ... ... ... ... ... ... 1903
English Catalogue of Books for 1903, The ... ... ... ... ... ... ... 1904
Epidemiological Society of London, Transactions of the Vol. XXII. 1903
Gazette des Hôpitaux ... ... ... ... ... ... ... ... ... ... ... ... 1903
Gazette des Hôpitaux de Toulouse ... ... ... ... ... ... ... ... 1903
Gazette hebdomadaire des Sciences médicales de Bordeaux Tom. XXIV. 1903
Gazette médicale de Paris ... ... ... ... ... ... ... ... ... ... ... 1903
Giornale internazionale delle Scienze mediche ... ... ... ... ... ... 1903
Glasgow Medical Journal, The ... ... ... ... ... ... ... Vol. LX. 1903
Hospitals—
Guy's Hospital Reports ... ... ... ... ... ... ... ... Vol. LVIII. 1904
Johns Hopkins Hospital Reports, The ... ... ... ... ... Vol. X. 1902
St. Thomas's Hospital Reports ... ... ... ... ... ... Vol. XXXI. 1904
Indian Medical Gazette, The ... ... ... ... ... ... ... Vol. XXXVIII. 1903
Intercolonial Medical Journal of Australasia ... ... ... Vol. VIII. 1903
International Medical Magazine ... ... ... ... ... ... Vol. XII. 1903
Johns Hopkins Hospital Bulletin, The... ... ... ... ... Vol. XIV. 1903
Journal d'Hygiène ... ... ... ... ... ... ... ... ... ... Vol. XXVIII. 1903
Journal of Balneology and Climatology, The ... ... ... (3) Vol. VII. 1903
Journal of Hygiene, The... ... ... ... ... ... ... ... ... Vol. III. 1903
Journal of Laryngology, The... ... ... ... ... ... ... Vol. XVIII. 1903
Journal of Mental Science, The ... ... ... ... ... ... Vol. XLIX. 190
Journal of Obstetrics and Gynæcology of the British Empire, The Vol. IV. 1903
Journal of Tropical Medicine, The ... ... ... ... ... ... Vol. VI. 1903

Journal of Tuberculosis, The... ... ... ... ... ... ... ... ... ... Vol. V. 1903

Laryngological Society of London, Proceedings of the ... (6) Vol. X. 1903

Laryngoscope, The ... ... ... ... ... ... ... ... ... ... ... Vol. XIII. 1903

Library Association Record, The ... ... ... ... ... ... ... ... Vol. V. 1903

Library Association Year-Book, The ... ... ... ... ... ... ... ... ... 1904

Library, The ... ... ... ... ... ... ... ... ... ... ... N.S., Vol. IV. 1903

Lucerne County Medical Society, Transactions of the ... ... ... ... 1903

Medical Age, The ... ... ... ... ... ... ... ... ... ... Vol. XXI. 1903

Medical Annual, The ... ... ... ... ... ... ... ... ... ... ... ... 1904

Medical Chronicle, The ... ... ... ... ... ... ... ... 4th Ser., Vol. V. 1903

Medical Examiner, The ... ... ... ... ... ... ... ... Vols. II.–IV. 1901–03

Medical News, The ... ... ... ... ... ... ... ... ... Vol. LXXXIII. 1903

Medical Press and Circular, The ... ... ... ... ... ... [Vol. CXXVII.] 1903

Medical Record, The ... ... ... ... ... ... ... ... ... ... Vol. LXIV. 1903

Medical Review, The ... ... ... ... ... ... ... ... ... ... ... Vol. VI. 1903

Mercy and Truth ... ... ... ... ... ... ... ... ... ... ... Vol. VII. 1903

Monthly Cyclopædia of Practical Medicine, The ... ... ... Vol. VI. 1903

Montreal Medical Journal, The ... ... ... ... ... ... ... Vol. XXXII. 1903

Nord médical, Le ... ... ... ... ... ... ... ... ... ... Tom. IX. 1903

Occidental Medical Times ... ... ... ... ... ... ... ... ... Vol. XVII. 1903

Odontological Society of Great Britain, Transactions of the

Vol. XXXV. 1903

Pennsylvania Medical Journal, The ... ... ... ... ... (8) Vol. VI. 1903

Post-Graduate, The ... ... ... ... ... ... ... ... ... Vol. XVIII. [1903]

Practitioner, The ... ... ... ... ... ... ... ... ... ... [Vol. LXXI.] 1903

Progrès médical, Le ... ... ... ... ... ... ... ... Tom. XVII., XVIII. 1903

Providence Medical Journal, The ... ... ... ... ... ... (9) Vol. IV. 1903

Publishers' Circular, The ... ... ... ... ... ... ... (12) Vol. LXXIX. 1903

Revue de Thérapeutique médico-chirurgicale ... ... ... ... ... ... 1903

Revue générale d'Ophtalmologie ... ... ... ... ... ... ... Tom. XXII. 1903

Revue hebdomadaire de Laryngologie ... ... ... ... ... Tom. XXIII. 1903

Revue pratique d'Obstétrique et de Gynecologie ... ... [Tom. XIX.] 1903

Rhode Island Medical Society, Transactions of the, (10) Vol. VI., Part 5 1903

St. Louis Medical and Surgical Journal, The, Vols. LXXXIV., LXXXV. 1903

St. Petersburger medicinische Wochenschrift ... ... ... ... ... ... 1903

Sanitary Record, The ... ... ... ... ... ... ... ... ... Vol. XXXII. 1903

Scottish Medical and Surgical Journal, The ... ... ... Vol. XIII. 1903

Swansea Rural District Council, Medical Officer's Annual Report on the Llandilo-Talybont Division of the, for 1903... ... ... ... ... 1904

Therapeutic Gazette, The ... ... ... ... ... ... ... ... Vol. XXVII. 1903

Treatment ... ... ... ... ... ... ... ... ... ... ... ... Vol. VII. 1903–04

Tuberculosis ... ... ... ... ... ... ... ... ... (7) Vols. I., II. 1899–1903

West London Medical Journal ... ... ... ... ... ... ... Vol. VIII. 1903

Wiener klinische Wochenschrift ... ... ... ... ... ... ... ... ... 1903

Zentralblatt für Chirurgie ... ... ... ... ... ... ... ... ... ... ... 1903

Zentralblatt für Gynäkologie ... ... ... ... ... ... ... ... ... ... 1903

Zentralblatt für innere Medicin ... ... ... ... ... ... ... ... ... ... 1903

### Local Medical Notes.

**University College, Bristol.**—Students of the College have been successful in the following examinations:—University of London *B.S.*: Walter C. Swayne, M.D. (Lond.). *M.B.* (Second Div.): S. R. Williams. *Intermediate M.B.*: C. E. K. Herapath, C. S. Rivington. *Doctor of Science*: John Herbert Parsons, M.B., B.S. (Lond.). Conjoint Board of the Royal Colleges of Physicians and Surgeons of England.—*Practical Pharmacy:* E. Christofferson, W. T. Clarke, E. J. Dermott, J. L. O. Tilley. *Biology*: E. J. D.

Haining, H. H. S. Templeton. *Chemistry:* E. E. Davies. *Anatomy and Physiology:* E. S. Goss, F. J. McQueen, H. T. Rossiter, R. G. Vaughan, F. T. Boucher, R. J. G. Parnell. *Medicine:* F. P. Hughes,* J. C. Wadmore,* S. C. Hayman. *Surgery:* F. H. Rudge.* *Midwifery:* R. O. Bodman, V. A. Crinks, L. N. Morris.—L. D. S. (Eng.). *Preliminary Science Examination:* H. T. Genge. *Final Examination*, Part I.: W. E. Boulter,* A. J. Mundy. Part II.: George H. Smith.*—L. S. A. *Surgery:* P. F. Howden, R. Reynalds.*—L.R.C.P. and S. (Edin.), L.F.P. and S. (Glasg.): J. Crétin.

**Bristol Royal Infirmary.**—*Assistant Dental Surgeon:* Charles A. Hayman, M.D., F.R.C.S.I., L.R.C.P.I., L.D.S.

**Bristol General Hospital.**—*Assistant House Physician:* C. A. Moore, M.R.C.S., L.R.C.P. *Casualty Officer:* F. H. Pickin, M.R.C.S., L.R.C.P. *Assistant House Surgeon:* A. R. Short, M.B., B.S., B.Sc. (Lond.).

**Royal Hospital for Sick Children and Women.**—*House Surgeon:* K. H. Beverley, M.R.C.S., L.R.C.P.

**Hospital Sunday Fund.**—We are indebted to Dr. Bertram M. H. Rogers, Secretary to the Committee which deals with the above Fund, for the information that the number of collections for the Fund were 327 in 1903, and 383 in 1904. There were thus 56 more collections in the present than in the preceding year. The amount collected was £1,720 3s. 8d. in 1904, and £1,565 4s. in 1903, showing an increase in the present year of £154 19s. 8d.

**Winsley Sanatorium.**—The annually-recurring fête or carnival is announced, under the name of "OPENAIRIA," to be held on June 29th and three following days. Its object this year is to raise money for the furnishing and equipment of the Winsley Sanatorium for consumptives. It can hardly be necessary to remind our medical colleagues that the Sanatorium is for the accommodation of patients, not from Bristol alone, but from all parts of the three counties of Gloucester, Somerset, and Wilts, and that the local committee may rightly look for the support of neighbouring districts, which will participate equally with Bristol in the benefits which it will undoubtedly confer on consumptives of limited means residing in this large district. The Right Hon. Lewis Fry, P.C.; Sir John Dickson Poynder, Bart., M.P.; the High Sheriff of Bristol (H. Greville Edwards, Esq.); and the Lord Bishop of Bristol have severally kindly consented to open the fête on successive days.

* Completes examination.

